# The Connection Between Sex as Self-Injury and Sexual Violence

**DOI:** 10.1007/s10508-023-02669-5

**Published:** 2023-08-28

**Authors:** Ludwig Hedén, Linda S. Jonsson, Cecilia Fredlund

**Affiliations:** 1 Faculty of Medicine and Health Sciences, Linköping University, Linköping, Sweden; 2 Department of Social Sciences, Marie Cederschiöld University, Stockholm, Sweden; 3 Department of Psychiatry in Linköping, and Department of Biomedical and Clinical Sciences, Linköping University, SE-581 83 Linköping, Sweden

**Keywords:** Sexual violence, Sexual abuse, Sex as self-injury, Nonsuicidal self-injury, Revictimization, DSM-5

## Abstract

Nonsuicidal self-injury (NSSI) is a suggested diagnosis recognized by DSM-5 as in need of further research. Recent studies conclude that sex as self-injury (SASI) and NSSI share similar functions, such as being used as emotional regulation. SASI has been associated with earlier sexual abuse, but the understanding of the association between sexual violence and SASI is still limited. The aim of this study was to further investigate the connection between sexual violence and the experience of SASI. How could SASI be related to sexual violence? The study used a qualitative design and was based on an anonymous questionnaire published on the websites of Swedish NGOs, providing help and support to women and youths, such as those in women’s shelters. In total, 139 informants with a mean age of 27.9 years (range 15–64 years) were included in the study. Three main themes were found: (1) Normalization of sexual violence and a shift in boundaries could be seen as consequences of earlier experiences of sexual violence leading to SASI; (2) SASI could escalate into sexual violence through increased need of emotional regulation, increased risk-taking, and risk of victimization; (3) SASI could be used to regain control of re-experiences, the body, sexuality, and shame after sexual abuse. In conclusion, a complex connection was identified whereby SASI could escalate into sexual violence, and earlier experiences of sexual violence could lead to SASI in a vicious loop. Hence, SASI should be seen as a risk factor for further victimization and sexual abuse.

## Introduction

### Sexual Violence

Sexual violence has been defined by the World Health Organization (WHO, 2002) as “any sexual act, attempt to obtain a sexual act, unwanted sexual comments or advances, or acts to traffic, or otherwise directed, against a person’s sexuality using coercion, by any person regardless of their relationship to the victim, in any setting, including but not limited to home and work.” Sexual violence is an umbrella term which includes a broad range of sexually violent actions such as rape within relationships, rape by strangers, unwanted sexual advances or sexual harassment, sex in return for favors, child sexual abuse, sexual abuse of mentally or physically disabled people, forced marriage, denial of right to use contraception, forced abortion, violent acts against sexual integrity such as genital mutilation, and sexual exploitation such as prostitution and trafficking (WHO, 2002). In this study, sexual violence will be used as a broad term that includes sexual abuse, sexual exploitation, child sexual abuse (CSA), sexual assault, rape, and sexual harassment.

Sexual violence is associated with poor psychosocial health and is associated with poor outcomes, including physical effects on both sexual and reproductive health, such as urinary tract infections, sexually transmitted infections, and chronic pelvic pain (Hailes et al., 2019; Maniglio et al., 2009). Effects on mental health include depression, post-traumatic stress disorder (PTSD), eating disorders, somatoform disorders, depression, anxiety, nonsuicidal self-injury, and suicide attempts (Chen et al., 2010; Hailes et al., 2019; Serafini et al., 2017). Sexual violence might lead to consequences among victims, such as compulsive sexual behavior, and increased risk-taking behaviors such as earlier age of sexual debut, prostitution, and alcohol and drug abuse (Hailes et al., 2019; Öberg et al., 2021; Ports et al., 2016; Scoglio et al., 2021; Senn et al., 2008; Slavin et al., 2020).

Victims of sexual abuse are at greater risk of sexual revictimization compared to peers without experience of sexual violence (Öberg et al., 2021; Ports et al., 2016; Scoglio et al., 2021). A cross-sectional, population-based study concerning sexual violence against women in Sweden found that sexual violence during childhood was associated with rape/attempted rape in adulthood. When sexual violence in childhood was combined with physiological violence, physical violence, or both, associations with adulthood sexual revictimization were even stronger (Öberg et al., 2021). Further studies, including informants exposed to CSA, found a 3.17 times higher risk of being exposed to sexual revictimization (Ports et al., 2016). Other factors associated with revictimization are childhood maltreatment, PTSD, emotional dysregulation, and maladaptive coping strategies (Scoglio et al., 2021).

### Self-Injurious Behaviors

Research concerning self-injurious behavior (SIB) has grown considerably during the past 20 years. There has been an ongoing debate on the definitions, conceptualizations, and classifications of SIB, since many different terms have been used (Hooley & St. Germain, 2014; Muehlenkamp, 2005; Nock, 2010). In the fifth edition of DSM-5, an attempt was made to establish a single concept to facilitate more uniform research by introducing nonsuicidal self-injury (NSSI), in contrast to suicidal behavior (American Psychiatric Association, 2013). NSSI usually refers to behaviors such as burning, scratching, cutting, biting, and self-hitting, which are self-inflicted injuries directed to the surface of the body with expectations of relieving negative feelings or thoughts, or to induce positive feelings (American Psychiatric Association, 2013; International Society of the Study of Self-Injury, 2021). However, the concept of NSSI is considered as in need of more research for it to be defined as a proper diagnosis according to DSM 5. Studies have found that sex could be used as a means of self-injury, but the behavior still lacks a common definition. In our study, sex as self-injury (SASI) was defined for the participants as: “to have repeatedly sought sexual situations that have caused you physical and/or mental harm and that have affected you in your life.” SASI is not commonly considered as part of NSSI, even though the two behaviors share common functions and could even replace one another with regard to the function (Fredlund et al., 2020; Jonsson et al., 2019). The research concerning SASI is still limited, and the behavior is mostly described in Swedish contexts even though studies have found it as a relevant concept in other cultures (Fredlund et al., 2017, 2020; Jonsson et al., 2019; Mann et al., 2022; Mellin & Young, 2022).

### Prevalence and Risk-Factors of Sex as Self-Injury

Few prevalence studies have been made concerning SASI, but in a national study of Swedish school students with a mean age of 18 years, 3.2% of girls and 0.8% of boys reported using sex to intentionally hurt themselves on any occasion (Fredlund et al., 2017). In a small pilot study of U.S. college students, 12% reported using sexual activity to self-injure, which included physical pain, reliving past trauma or self-harm, suicidal ideation, confirmation through feeling needed or wanted, proving self-worth, or pleasing (Mellin & Young, 2022). The most common function of both SASI and NSSI is emotional regulation, such as to stop bad feelings, to relieve feelings of being numb or empty, to feel something (even if it is pain), to feel in control, to get attention, to self-punish, or to express body hatred (Jonsson et al., 2019; Taylor et al., 2018). However, interpersonal functions such as receiving positive or negative confirmation is more commonly found in SASI compared with NSSI (Jonsson et al., 2019). SASI could be used as a means to cope with anxiety and negative emotions, to self-punish, and to receive positive or negative confirmation, and may be manifested in various ways, such as selling sex, engaging in sexual contacts without sexual attraction or desire, exposing oneself to physical violence and pain in sexual situations, causing injury to one’s own genitals, or repeating earlier sexual abuse (Fredlund et al., 2020).

NSSI has been associated with a higher risk of subsequent suicidal behavior, including suicide ideation (OR = 2.8), suicidal plans (OR = 3.0), and suicide attempts (OR = 5.5) (Kiekens et al., 2018). Strong associations have been found between SASI and suicide attempts but also other risk-taking or self-destructive behaviors, such as sexual risk-taking, drug or alcohol abuse, and eating disorders (Fredlund et al., 2017; Zetterquist et al., 2018). NSSI has been associated with multiple causal factors, including interpersonal stressors, neurobiological background, emotional dysregulation, and adverse experiences in childhood (Brown & Plener, 2017; Serafini et al., 2017; Wolff et al., 2019).

### Sex as Self-Injury and Sexual Violence

In a questionnaire-based study of a representative national sample of Swedish third-year high school students, 75% of participants with self-reported experience of SASI had been previously exposed to sexual abuse (Fredlund et al., 2017). When comparing adolescents with SASI to adolescents with NSSI, SASI was found to be associated with penetrative sexual abuse, higher numbers of sexual partners, and a greater level of self-reported trauma symptoms, such as dissociation, post-traumatic stress, and traumatic sexual concerns. The group with both NSSI and SASI were identified as a particularly vulnerable and burdened group regarding experience of abuse, presence of risk-taking behaviors, and impaired psychosocial health (Zetterquist et al., 2018). Also, in more qualitative research, earlier sexual abuse has been described as an underlying reason to start using sex as a means of self-injury (Fredlund et al., 2020).

In conclusion, sex could be used as a means of self-injury with comparable functions to NSSI, including the function of emotional regulation. Differences regarding risk factors between the two behaviors have especially been found to be earlier experiences of sexual abuse and trauma symptoms more commonly seen in SASI. There is today a lack of understanding of how SASI could be related to sexual violence.

### Aim of the Study

The aim of this study was to get a deeper understanding of how sexual violence and SASI are connected. The study will emanate from the following research question: How can SASI be understood in relation to sexual violence? This was done first by listening to the thoughts and experiences of the study participants, and subsequently, by analyzing the data using thematic analysis.

## Method

### Participants and Procedure

This study was based on an anonymous and open-ended questionnaire that was published on the websites of Swedish non-governmental organizations (NGOs), such as women’s shelters, young women’s shelters, and other organizations that aim to provide help and support to women and youths. The organizations were chosen due to their work with vulnerable women and youths, with the expectation of finding participants with experience of SASI, based on previous research associating SASI with earlier sexual, physical, and emotional abuse (Fredlund et al., 2017). In total, 37 NGOs from all parts of Sweden published the link to the questionnaire in the period of December 2016–April 2017 with the following wording: “Do you have experience of SASI, and are you over 15 years of age? Do you want to participate in an anonymous questionnaire-based study to increase understanding of SASI and improve help and support? Click on this link.” The questionnaire was answered anonymously and SASI was defined in the questionnaire as: “To have repeatedly sought sexual situations that have caused you physical and/or mental harm and that have affected you in your life.”

To be included in the survey, participants had to be older than 15 years of age and have had experience of SASI. The lower age limit was set at 15 because no consent from a legal guardian is required for studies in Sweden when participants are older than 15 years (The Ministry of Education & Cultural Affairs, 2003).

The survey was completed by a total of 199 informants, of whom 190 were women, four were men, and four were non-binary, and one person did not answer the question regarding gender. There was no explicit question concerning experience of sexual violence, but 139 informants reported experiences of sexual violence that was found as relevant for the index question; hence, 139 informants contributed to the analysis. The age varied between 15 and 64, with a median age of 27 and a mean age of 27.9 (SD = 9.3). In total, 82.9% of the informants reported that their first experience of SASI was during adolescence, between the ages of 12 and 19, while 4.0% reported being younger than 12 and 10.6% reported being 20 or older.

### Measures

The questionnaire included twelve open-ended questions about subjects including participants’ personal data, motives, experiences of SASI, and experiences of help and treatment—see Appendix 1. The questionnaire was tested in a pilot study comprising five informants. Artologik Survey and Report, a web-based survey program acquired by Linköping University, was used to collect the answers for the questionnaire (Artologik, 2021).

### Data Analysis

The data were analyzed by using thematic analysis, following the six steps proposed by Braun and Clark (2006). Initially, to gain familiarity with the data, all material from the survey was first read thoroughly several times by the two authors, LH and CF, followed by the step of generating initial codes. The text was read word by word, with the purpose of identifying material in the questionnaire that answered the research question of the study, and subsequently collecting and coding the interesting features. After coding relevant segments, the study material was read through a fourth time with a focus on produced codes that were organized into initial themes and subthemes. These themes were further assessed in relation to both coded extracts and the whole data set, and a thematic map was generated in discussion between the two authors, LH and CF. In the further step, through discussion between the authors, themes were more specifically defined and named to refine and evaluate the overall story of the analysis. In the final phase, quotations from informants confirming the recognized themes and subthemes were identified and chosen.

In the analysis process, the first author (LH) took the lead in the coding of the text using a codebook with definitions. With regard to triangulation, the last author (CF) coded part of the study material to enable a discussion and comparison of coding categories and to facilitate the forming of themes that were made in discussion in the research team. Themes were discussed and examined regarding internal organization and fitted with chosen codes and quotations. This procedure was completed during continual discussion within the research group until saturation was achieved. The quotations were, lastly, translated into English by the author with the support of professional translators, since the survey was originally conducted in Swedish. The NVivo 12 software was used as support for the analysis, to keep track of the thematization and citations used. In Table 1, the number of participants reporting the main findings is calculated to give the reader a feeling of the recurrency of themes and subthemes.

**Table 1:** Main findings found in the connection between self-injury and sexual violence

	*n* = 139	%
1. Normalization of sexual violence and shift in boundaries		
Normalization of sexual violence	61	43.9
Difficulties in setting boundaries	32	23.0
2. Escalation of violence		
Increased need of emotional regulation	66	47.5
Increased risk-taking	43	30.9
Increased risk of victimization	23	16.5
3. Regaining control after sexual abuse		
Regaining control of re-experiences	20	14.4
Regaining control of the body and sexuality	18	12.9
Control of shame	11	7.9

## Results

The study found a complex connection between SASI and sexual violence, where SASI could lead to sexual violence, and experiences of sexual violence could lead to SASI, creating a vicious circle. As seen in Table 1, in total, three main themes, together with eight subthemes, were identified describing this connection: (1) normalization of sexual violence and a shift in boundaries; (2) escalation of violence; and (3) regaining control after sexual abuse.

### Normalization of Sexual Violence and a Shift in Boundaries

Normalization of sexual violence was described as one reason for engaging in SASI by informants with a history of sexual abuse, especially that related to CSA. Experience of sexual abuse was described as affecting the informants’ norms on sex and sexuality. The boundaries of normal sexuality had been shifted toward including more violent and abusive acts, often due to the feeling of not deserving better. The perpetrators, together with an absence of appropriate reactions from people close to them, made victims believe that the sexual abuse they were subjected to, and self-destructive sex, were normal. Self-destructive sex was believed to be a way to show affection and that it was the duty of a woman to please men. One woman, aged 20, wrote:

After I came out of a destructive relationship in which my then-boyfriend repeatedly raped me, I felt like I wasn’t worth anything else [other than SASI]. He had made my body something public. Something that anyone had access to. /…/ I thought that was just what it was like to be a girl. Everything in me was so fucking gloomy. Sex was the only thing that made it quiet inside for a while. Because that was the only time I felt normal. Getting guys’ confirmation and appreciation became a fuel that triggered me to continue to have lots and lots of sex, while at the same time everything inside me became worse. I met guys through various dating apps. Did nothing for my own enjoyment. Always for them. Always to silence the one inside of me that hurt so much. (Woman, 20 years old. Ax194).

Informants described a normalization of sexual violence after having previously been subjected to a long period of ongoing abuse in their childhood, in adolescence, or in long-term abusive relationships during adulthood. Some informants described that this type of sexual encounter was the only type of sex they felt comfortable with, and the kind of sex that they deserved. One informant wrote:

I felt like I didn’t deserve anything else [other than self-destructive sex]. I tried to have a relationship with a guy who was really decent and cared more about me than any other guy I had before. But it scared me. He was someone who would never hit me or be forceful to me when we had sex; instead, there was love and tenderness in sex and it made me so scared and disgusted. It got too tough to handle; I couldn’t stand it. I don’t deserve good love and can’t handle it. It makes me feel even more vulnerable and threatened. I just keep on hearing nasty words about myself in my head and can’t believe anyone when they say that they like me. Because I believe that no one likes me for who I am, only to get to my body. (Woman, 20 years old. Ax83).

Difficulties setting boundaries with sexual partners was described in relation to SASI. Informants reported being unable to deny partners sex and being incapable of putting their own wishes first. Informants argued that their active role in seeking sex through SASI lessened their own feeling of having a right to deny sexual activities they did not want to engage in. Informants describe feeling that they deserved any pain accompanied with SASI.

I had a secret “fuck-buddy” relationship with my sister’s best friend. I really had no desire to have sex with him, and many times it hurt terribly. But while we had sex, I used to think that it was right for me, that it was the only thing I was capable of, that I deserved the pain. I did things even though it felt disgusting and felt some sort of defiance in what I did. I dared to do the things others had forced me to do*.* (Woman, 30 years old. Ax186).

The informants list engaging in activities such as anal sex, oral sex, fisting, violent sex, and humiliating sex during SASI, even though they did not want to. After having once agreed to—or, at least, not actively having said no to, it became even harder to refuse unwanted sex in future sexual encounters, and their boundaries of what was acceptable in sexual situations shifted. The experience of crossing their own boundaries further bred self-hatred, disgust, and shame, even though the informants were the ones who were subjected to abuse.

I’ve had sex with guys I did not want to have sex with, but mostly I’ve agreed to do things I didn’t really want to do. I agreed to have anal sex several times with a guy even though it hurt so much one time that I started sweating and couldn’t help crying. And I used quite a lot of lubricant to hide that I wasn’t wet. But at the same time, I continued to meet both him and other guys. I always felt bad when I met them, and even worse afterwards, but at least I didn’t feel so much at all during the sex. (Woman, 21 years old. Ax139).

Informants report thinking that it was their duty to satisfy their sexual partner, to agree to all wishes, and that this would make them feel good. Whatever maltreatment informants received, they felt they deserved it.

One woman wrote:

For a long period (five years), I used to search for situations where men could or would hurt me in sexual contexts. I tried to destroy myself, my body, and my emotions by focusing on nothing but the men, what they wanted from me, and satisfying them. The men I sought out became increasingly violent and aggressive, wanted to “play” rape, exploit me, and humiliate me. (Woman, 28 years old. Ax63).

### Escalation of Violence

Increased need of emotional regulation was seen, triggered by poor mental health, including depression, anxiety, and self-hatred, as the primary underlying motive for SASI. SASI could fulfill the function of emotional regulation and getting attention, affection, and confirmation. Irrespective of the initial motive, SASI could confirm and reinforce the user’s negative self-image and further add to their feelings of self-hatred and low self-esteem, making them feel even worse. SASI created a vicious loop where the violence, humiliation, and abuse to which informants were exposed during SASI increased their self-hatred and negative emotions. To cope with these exacerbated feelings, and obtain the same relieving emotional regulatory effect, increased self-destruction and violence through SASI were needed, leaving informants feeling even worse. One informant wrote:

It [SASI] started in situations involving alcohol use. I sort of happened to end up in situations where sex was expected, and it escalated from there. Later on, I started looking for situations where I could expose myself to this self-harming behavior. It then required increasingly dangerous situations to ease my anxiety and I ended up in questionable situations a few times*.* (Woman, 24 years old. Ax101).

SASI could escalate into sexual violence through *increased risk-taking* and pushed limits of acceptance in sexual situations*.* For some informants, what began as casual sex without attraction, ended up in sexual exploitation and rape. In some cases, informants describe pushing their own sexual limits, engaging in sex with more and more abusive sexual partners. In other cases, they describe their limits actively being pushed by their sexual partners and them accepting it as a means of self-injury. One informant wrote:

Actually, it started a long time ago with [sexual] abuse when I was little, plus physical abuse. It has given me a fear of intimacy that still affects my life. But since I had pressure from those around me and from healthcare, I was desperate for a self-harming behavior that was invisible or left no scars. And through the internet I started to find guys, but it was not enough, so I started to sell myself for more abasement. When I got paid, I lost all the rights to my body and even the possibility to say no. As a further step in this I agreed to unprotected intercourse if there was a desire for this. I still get suicidal thoughts and anxiety today if someone just touches me. (Woman, 30 years old. Ax27).

When I was younger, it [SASI] was mostly about things one could do over the internet–webcam shows, for example–but when I moved away from home, I was firstly in extremely risky sexual relationships, and then started having sex for payment. (Woman, 21 years old. Ax128).

In this loop, informants were subjected to increased risk of victimization. In the search for emotional regulation through self-destruction, informants became easy targets for violent men. This risk exposure entailed many situations where the vulnerability of the informants, resulting from their poor mental health and their search for self-harm, was exploited by offenders. These risky situations could involve exposure to violent/abusive men, sexual situations involving humiliation, selling sex for money or drugs, and sexual interaction related to substance abuse, including alcohol and other drugs. One informant wrote:

I allowed everyone to exploit me. Had no control. Exposed myself to dangerous, risky situations where I allowed people to have sex with me, sometimes several at the same time, to injure myself in a destructive way. I was worth nothing and it was only when I had this verified (though assaults and exploitation) that I found peace. For a short while. I think I also felt it was easier to hate them [rather] than to hate myself for a while. (Woman, 27 years old. Ax144.)

### Regaining Control After Sexual Abuse

To regain control of re-experiences of earlier sexual abuse was described by the informants as an underlying function of SASI. Many informants with experiences of sexual abuse reported it being a starting point for their engagement in SASI. Experiences of sexual abuse had caused them psychological sequela, such as long-lasting negative emotions with anxiety, numbing, and re-experiences through post-traumatic symptoms. This included constantly reliving their traumatic event through flashbacks and nightmares. Informants described that SASI could function as a method to cope with these negative emotions, which momentarily reduced anxiety. Engaging in SASI was a way to escape flashbacks by, instead, re-experiencing previous abuse in real life, through which they aimed to regain control of the experiences. One woman, aged 22, wrote:

I didn’t understand that my behavior was destructive. It was like a compulsion, an addiction. Worse than other types of self-harming behaviors I had. Lots of shame, and it was used to punish myself. To manage stress and emotions. To get the abuse “out of my head.” It was easier when it really happened than when it was just vague memories and a lot of fear and shame that no one knew about, that I had no words for, and that I could not shake off. (Woman, 22 years old. Ax150).

To escape flashbacks, some informants sought casual sexual contacts and partnerships with violent men to self-injure, but informants were often revictimized and exposed to similar violence to that of which they had previously been victims. This included rape, strangulation, humiliation, beatings, being held down, or being penetrated with harmful objects. Some informants described self-injuring by their own hand during flashbacks through compulsive masturbation, inserting harmful objects into their genitals, self-strangulation, self-beatings, and cutting their genitals. Some informants self-injured both by their own hand and through sexual partners. Informants describe this search for abuse as a better alternative to reliving through flashbacks. The real experience of violence and abuse was easier to cope with. One 22-year-old woman wrote:

I got anxiety/shame/flashbacks. The worst was the vague flashes that I did not remember completely, but instead only felt in my body. I couldn’t handle it. Wished the memory would come “for real” instead. So, then I used to…well. Simply do what Dad used to do when I was little. I hit myself. I stuck things in so it hurt and bled or simply cut myself down there. Strangled myself, etc. Or I did other degrading things. Sometimes not as violently and without physical harm, but it felt like it was Dad who did it and I didn’t want him to. Sometimes I talked to mean men online at the same time. I cried, said no, wanted it to stop, but continued anyway. It’s like I’m abusing myself. The perpetrator did not even have to be there anymore. (Woman, 22 years old. Ax150).

To regain control of their own body and sexuality, after earlier abuse, was described by the informants as an important function of SASI. Informants describe how being violated made them feel like their body and/or sexuality no longer belonged to them. SASI brought a sense of regaining control of their body. Since informants played an active part in seeking sexual situations, they hoped that they would no longer be victims of sexual abuse. One informant wrote:

I scheduled meetings with older men through the internet to have sex. I didn’t care what they did to me. Didn’t enjoy it at all but gained the feeling of deciding when the abuse will happen. That if I’m in control of when things happen and with whom, I can never be raped again. And if someone hit me, I thought I deserved it. Afterward, however, I got anxious about what I had done no matter what. Relief from anxiety turned into anxiety*.* (Woman, 26 years old. Ax165).

To regain control of shame after sexual abuse was another reason for SASI–this by daring to have new sexual interactions and to stop feeling ashamed of having been abused. This specifically applied to those who experienced shame after rape, and to those who had been shamed through being labeled as promiscuous by others after engaging in sex. Sexual abuse was also described by informants as inducing the feeling of being abnormal. Engaging in sex to feel normal was a way for informants to regain control of their sexuality. This can be exemplified by the testimonies of informants:

I think it [SASI] started after the first time I had sex, when I was raped at a party. I didn’t want to feel “ashamed,” so I started having sex with everyone I could. I didn’t care that it wasn’t nice; I just wanted to have sex for the kick*.* (Woman, 23 years old. Ax193).

## Discussion

Even though SASI is still not a well-described phenomenon in the literature, existing studies point in the direction that SASI is associated with experiences of sexual abuse both prior and during SASI (Fredlund et al., 2017, 2020; Jonsson et al., 2019; Zetterqvist et al., 2018). This has been confirmed in the current study. The aim of this study was to understand how SASI and sexual violence are connected and investigate how sexual violence could be related to the experience of sex as a means of self-injury. This study found a connection between SASI and sexual violence where SASI could escalate into sexual violence, and experiences of sexual violence could lead to SASI. The main findings of the study will be discussed below.

First, our study indicates that SASI could, through escalating violence, predispose repeated abuse and sexual revictimization. Studies conclude that victims of sexual abuse are at a greater risk of sexual revictimization (Miron & Orcutt, 2014; Öberg et al., 2021; Ports et al., 2016; Scoglio et al., 2021). This is often explained by a combination of psychological distress, coping strategies, and sexual behavior. A longitudinal study of American college women concluded that a history of childhood physical or emotional abuse was associated with prospective adult sexual assault, while a history of childhood sexual abuse was associated with sexual revictimization in adolescence. Furthermore, it predicted a likelihood of risky sexual behavior (Miron & Orcutt, 2014). This study indicates that SASI could be, through normalization of violence, increased risk-taking, need of emotional regulation, and increased risk of victimization, be connected to revictimization and further sexual violence. A vicious loop was described with SASI that added to informants’ negative self-image and poor mental health, further increasing their self-hatred and negative emotions, and making them increase their need of emotional regulation, including more severe violence and risk-taking through SASI. As such, SASI puts particularly vulnerable people in situations where the violence and risks could escalate, and they become easy targets of victimization. Hence, SASI should be seen as a risk factor for being exploited and abused, but more research is needed.

Second, questions could be raised over whether SASI even should be viewed as part of the concept of sexual violence: sexual violence directed toward oneself. As in sexual violence, a range of experiences are described related to SASI, from less violent sex including masturbation and sex with different partners, to very severe sexual violence including humiliation and sexual exploitation. Poor mental health, need of emotional regulation, normalization of sexual violence, a shift in boundaries, feelings of shame, and a misinterpretation of consensual responsibility are all factors that make a person with SASI vulnerable to exploitation by other persons who may take advantage of the vulnerable position. Legally, in accordance with the 2018 Swedish Consent Act, the law concludes that all sexual acts should be grounded in voluntariness and mutual consent, which means that a perpetrator could be convicted of sexual abuse without having carried out acts of violence or made threats. The Swedish Consent Act also specifies situations where consent cannot be given and consequently sex cannot be voluntary. This includes sexual actions after violence or threat, sexual acts with children (younger than 15 years of age) and exploiting a person in a position of dependency to the perpetrator. Also exploiting a person in a particularly vulnerable situation, including someone unconscious, someone affected by alcohol or drugs, or by sickness, disability, or psychiatric disorders; or if the person, due to the circumstances, is in a particularly vulnerable position (The Swedish Criminal Code, 2022). As has been discussed above, it could be argued that a person with SASI is in a particularly vulnerable position that could be recognized and exploited by offenders. Our experience is that today, instead of considering this as rape, the judgment in court could be in favor of the perpetrator instead of the victim in cases that include a person in a vulnerable position due to SASI. This needs to be investigated in further research by researchers with more knowledge of the law system. To view SASI in the context of sexual violence is important in terms of the legal processes, while understanding the function of SASI is important to provide proper help and support for the behavior, and to help victims emerge from a position of vulnerability, instead of just being seen as victim.

Third, the study concludes that SASI could function to help victims cope with previous experiences of sexual abuse. Informants described engaging in SASI while aiming to regain control of re-experiences of earlier sexual abuse and to take back control of their own body/sexuality after being sexually victimized. Informants described that it was easier to cope with real experiences through SASI than re-experiences such as flashbacks, due to the sense of control it entailed. Normalization of sexual violence after earlier experiences of sexual abuse, and a shift in boundaries, were also described, especially in relation to experiences of sexual abuse during childhood. The long-term effect of sexual abuse in relation to sexual function has been described in several studies in the last few decades (Barbara et al., 2022; Dworkin et al., 2017; Finkelhor & Browne, 1985; Hailes et al., 2019; O’Callaghan et al., 2019; Van Berlo & Ensink, 2000). As early as 1985 Finkelhor and Browne presented the traumagenic dynamics model, including traumatic sexualization, betrayal, powerlessness, and stigmatization to describe psychological damage caused by CSA, a model that is still used and frequently cited today. Traumatic sexualization refers to the process of how a victim’s sexuality is formed and developed in an inappropriate and dysfunctional manner due to CSA. The effects of abuse connected to traumatizing sexualization include developing compulsive or abusive sexual behavior, being sexually exploited, aversion to sex and intimacy in general, and confusion about sexual norms, standards, and sexual identity. The dynamics of betrayal describe a situation where the victim discovers that someone whom they depend on has done them wrong, which might lead to the need to regain trust and security, and the disinclination toward sex and intimacy. Powerlessness entails the abuser entering the victim’s personal space and body against their, will, creating anxiety and fear and impairing the victim’s sense of self-worth. This might explain the need to regain control through sexually dominating others or by re-enactment, such as seeking control by re-experiencing the vulnerability connected to abuse. Stigmatization could explain why victims of abuse describe considerable guilt and shame linked to their abuse, and of being stigmatized by others around them. This might lead to involvement in drug or alcohol abuse, prostitution, criminal activity, self-destructive behavior, and suicide. This model might serve as a foundation when attempting to understand how sexually abused victims develop SASI. This model also needs to be updated with the new research and the knowledge that we have gained in the last 30 years, including the knowledge concerning SASI.

### Limitations

The study used an online questionnaire, which entailed both advantages and disadvantages. It gave us a large sample of answers, possibly including participants that would not commit to interview studies. This method was chosen after taking into account the sensitive topic it included and with the expectation of providing a diversity in terms of both participants and answers. However, it did not give us opportunity to ask follow-up questions, and the informants’ responses might be shorter compared to responses in face-to-face interviews. Also, due to the recruitment process, most informants had a female gender identification; this means that it could not be generalized to men and people with non-binary identification.

An important limitation was the fact that the study aimed to investigate the concept of SASI but did not include specific questions concerning experience of sexual violence. This means that quantitative estimations on the recurrence of themes and subthemes should be received as an opportunity to get an overview of the data, but since this was a qualitative study, it did not provide the opportunity to make quantitative conclusions. Triangulation has been made to increase the trustworthiness, but some statements are vague, and calculation of citations could therefore be misleading.

### Conclusions

In conclusion, earlier experiences of sexual abuse could lead to SASI due to normalization of sexual violence and shift in boundaries. SASI could be used with the aim to regain control of re-experiences, body and sexuality, and shame after being sexually victimized. SASI could predispose repeated abuse and sexual revictimization due to increased need of emotional regulation, escalation of sexual risk-taking and violence, and increased risk of victimization. SASI should be seen as a risk factor for being exploited and abused. This study suggests that SASI might be viewed as one entity of sexual violence that is directed toward oneself, facilitating victimization and exploitation of the person. Due to the poor mental health and function of emotional regulation, SASI may increase the vulnerability of victimization of the person, which is why it needs to be addressed in society, the judicial system, and health care, but also, more research is needed. Understanding the underlying function of the behavior is important regarding health care, help, and support, and the association with sexual violence is important in relation to the legal process.

## Data Availability

That data could not be shared since it was not included in the ethical approval.

## Appendix: Study Questionnaire

**Table:**

1. How old are you?
2. What is your gender identity? E.g. woman, man, non-binary?
3. What do you usually do to cope with negative feelings or occurrences?
4. Describe your experiences of sex as self-injury
4a. In which way did you have sex as self-injury?
4b. How did it start?
4c. How old were you when you had sex as self-injury? Do you still have it?
4d. What made it continue?
4e. Describe a typical occasion when you had sex as self-injury, what happened?
4f. Think of a typical occasion. If this happened with another person, what relationship did you have to the other person and what was his/her age and gender?
4g. If you have stopped, what made you stop?
5. What experiences did you have of help and support when you had sex as self-injury?
6. What did you want in regard of help, support and treatment from healthcare or other organizations working with help and support when you had sex as self-injury?

## Code Availability

Codes will be shared on request.
